# Plant-Derived Bioactive Compounds and Potential Health Benefits: Involvement of the Gut Microbiota and Its Metabolic Activity

**DOI:** 10.3390/biom12121871

**Published:** 2022-12-13

**Authors:** Xinyu Chen, Shifeng Pan, Fei Li, Xinyu Xu, Hua Xing

**Affiliations:** 1 College of Veterinary Medicine, Yangzhou University, Yangzhou 225009, China; 2 Jiangsu Co-Innovation Center for Prevention and Control of Important Animal Infectious Diseases and Zoonoses, Yangzhou University, Yangzhou 225009, China; 3 Department of Animal Science, Washington State University, Pullman, WA 99163, USA; 4 Guangling College of Yangzhou University, Yangzhou University, Yangzhou 225009, China

**Keywords:** plant-derived bioactive compounds, gut microbiota, interactions, health benefits, feed additives, antibiotic substitute

## Abstract

The misuse and abuse of antibiotics in livestock and poultry seriously endanger both human health and the continuously healthy development of the livestock and poultry breeding industry. Plant-derived bioactive compounds (curcumin, capsaicin, quercetin, resveratrol, catechin, lignans, etc.) have been widely studied in recent years, due to their extensive pharmacological functions and biological activities, such as anti-inflammatory, antioxidant, antistress, antitumor, antiviral, lowering blood glucose and lipids, and improving insulin sensitivity. Numerous studies have demonstrated that plant-derived bioactive compounds are able to enhance the host’s ability to resist or diminish diseases by regulating the abundance of its gut microbiota, achieving great potential as a substitute for antibiotics. Recent developments in both humans and animals have also highlighted the major contribution of gut microbiota to the host’s nutrition, metabolism, immunity, and neurological functions. Changes in gut microbiota composition are closely related to the development of obesity and can lead to numerous metabolic diseases. Mounting evidence has also demonstrated that plant-derived bioactive compounds, especially curcumin, can improve intestinal barrier function by regulating intestinal flora. Furthermore, bioactive constituents can be also directly metabolized by intestinal flora and further produce bioactive metabolites by the interaction between the host and intestinal flora. This largely enhances the protective effect of bioactive compounds on the host intestinal and whole body health, indicating that the bidirectional regulation between bioactive compounds and intestinal flora has great application potential in maintaining the host’s intestinal health and preventing or treating various diseases. This review mainly summarizes the latest research progress in the bioregulation between gut microbiota and plant-derived bioactive compounds, together with its application potential in humans and animals, so as to provide theoretical support for the application of plant-derived bioactive compounds as new feed additives and potential substitutes for antibiotics in the livestock and poultry breeding industry. Overall, based on this review, it can be concluded that plant-derived bioactive compounds, by modulating gut microbiota, hold great promise toward the healthy development of both humans and animal husbandry.

## 1. Introduction

Antibiotics play an important role in ensuring the healthy development of both humans and animal husbandry, and have promoted the rapid recovery of the livestock and poultry breeding industry. In the past 70 years, the use of therapeutic and growth-promoting antibiotics has increased dramatically throughout the world. In the United States, the annual use of antibiotics is up to 16 million kg, about 70% of which is mainly used in animal husbandry and aquaculture [1]. In China, antibiotics used in aquaculture account for 46.1% of the total annual production, so as to ensure adequate aquaculture production. The misuse or abuse of disease-controlling and growth-promoting antibiotics together with their residue have raised significant concerns recently because of the potential serious threats to the health and safety of humans and the sustainable development of animal husbandry. Therefore, gradually reducing or even prohibiting the use of antibiotics and other drugs as additives to produce nonresistant livestock and poultry products is an inevitable trend for the development of the livestock and poultry industry worldwide. In addition, with the continuous improvement of consumer safety awareness, society’s demand for “nonresistant” livestock and poultry products is growing daily, thus, there is an urgent need to find alternatives to antibiotics. Moreover, Announcement No. 194 of the Ministry of Agriculture and Rural Affairs of the People’s Republic of China also requires that, beginning in 2020, the addition of antibiotics (except Chinese herbal medicine) to feed should be completely prohibited to reduce the harm caused by the misuse or abuse of antibiotics. Therefore, in the post-antibiotic era, to develop a new type of feed additive, which is drug free, nontoxic, and residue free that can replace antibiotics to improve the production performance of livestock and poultry, has become a research hotspot for scholars throughout the world. Under these circumstances, plant-derived bioactive compounds have become the first choice of antibiotic substitutes because of their unique advantages, such as rich resources, comprehensive functions, good biological safety, and various nutritional values, low toxicity and few side effects, less resistance, and low residue. Abundant evidence has shown that plant-derived bioactive compounds are able to promote the intestinal health of hosts by regulating the structure and abundance of intestinal microbiota and metabolites of bacterial flora. Moreover, as a very important part of the host, intestinal microbiota and its metabolites are involved in several physiological functions of the host by regulating various endocrine, neural, and immune pathways, including digestion, energy metabolism, and inflammation [2], thus playing remarkable roles in maintaining the health of their hosts.

Overall, gut microbiota perform some basic functions in the immunological, metabolic, structural, and neurological landscapes of the host’s intestine and whole body, though it is dynamic. An in-depth understanding of gut microbiota function has led to some very exciting developments in therapeutics, such as prebiotics, probiotics, drugs and fecal transplantation leading to improved health. In particular, plant-derived bioactive compounds can regulate intestinal flora and have great application potential in maintaining host intestinal health and preventing or treating numerous diseases.

## 2. Gut Microbiota

### 2.1. Biological Functions of Gut Microbiota

As the largest immune organ in the mammalian body, the intestinal tract comprises cells from nonhemopoietic and hemopoietic origin. It is also a dwelling (mainly located in the small bowel and colon) for trillions of microbes collectively known as the gut microbiota, including bacteria, archaea, fungi, and viruses [3,4]. The gut microbiota is a central regulator of host metabolism and can be considered a crucial “organ” of the host body because of its role in the maintenance of the balance between health and diseases. Numerous studies both in humans and animals have demonstrated that the gut microbiota has evolved along with their hosts and is an integral part of the host body, which is acquired at birth, develops in parallel as the host develops, and maintains its temporal stability and diversity through adulthood until death. These large and diverse intestinal microorganisms are always in a dynamic balance with the host, which is critical to stabilize the physiological functions and health of the host organism and can positively or negatively regulate its health [5]. However, intestinal microbiota composition is not constant, and the imbalance between intestinal microbiota and host is also considered to be one of the important factors inducing numerous diseases.

Intestinal microbiota can also benefit the host by transforming dietary nutrients into bioactive metabolites and play a role in maintaining gastrointestinal homeostasis together with the resident microbiota. In human and animals, gut microbiota ferments dietary nondigestible carbohydrates into short-chain fatty acids (SCFA), which are the major metabolites of intestinal microbiota, including acetate, propionate, and butyrate. Recent findings show that SCFAs, and in particular butyrate, also have important intestinal and health-modulatory functions. The different SCFA-producing intestinal microbiota has significant different potential in forming SCFAs, among which *Firmicutes* are the main microbiota producing butyrate [6]. In addition, other intestinal butyrate-producing bacteria include members of *Ruminococcus*, *Clostridium*, *Eubacterium,* and *Coprococcus* genera [7,8], while acetate is mainly produced by *Bifidobacterium* [9]. Additionally, acetate and propionate can also be formed by *Akkermansia muciniphila* [10]. Furthermore, different intestinal microbiota have interaction in the process of producing SCFAs; for example, *Bacteroides theiotaomicron* produces acetate, which can be further used by *Eubacterium hallii* to produce butyrate [11]. From these results, it can be concluded that SCFAs play a key role in providing energy sources [12], enhancing the integrity of the intestinal epithelial barrier [13], promoting the generation of regulatory T cells, and maintaining homeostasis in the host intestine [14], so as to strengthen the gut barrier functions as well as host health.

### 2.2. Main Factors Affecting the Composition of the Animal Gut Microbiota

**Figure 1:**
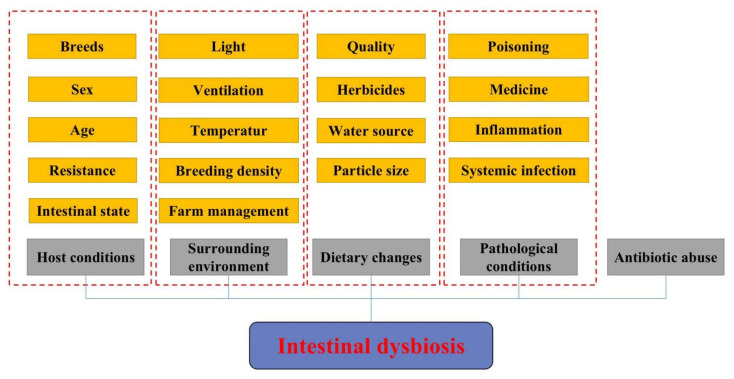
Main factors affecting the composition of animal gut microbiota.

The unbalanced symbiotic relationship among intestinal microbiota and the animal host and intestinal microbiota is called intestinal dysbiosis. The occurrence of this phenomenon is related to numerous factors, such as host conditions, surrounding environment, dietary changes, pathological conditions, antibiotic abuse, etc. (Figure 1). Host conditions mainly include different breeds (such as broilers and laying hens), sex, age, development level of the immune system, intestinal morphology, and intestinal movement. The environmental factors mainly include farm management level, breeding density, ventilation, light conditions, temperature control, etc. From the perspective of diet, the quality, change, particle size, and water source of the feed are extremely important to the intestinal microecology, and the antinutritional factors, heavy metals, toxic substances, bacterial toxins, herbicides, and antibiotics in the feed can all lead to the destruction of the intestinal microbiota, resulting in local inflammation, systemic infection, and even the emergence of typical poisoning symptoms [15]. In addition, pathological changes in diseases and drugs used in disease treatment, such as antibiotics, probiotics, prebiotics, and plant-derived active ingredients, can also lead to alterations in the intestinal microbiota.

### 2.3. Relationship between Gut Microbiota and Numerous Diseases

The gut microbiota is now considered as one of the key elements contributing to numerous diseases, including obesity, type 2 diabetes, hepatic steatosis, intestinal bowel diseases, and several types of cancer, by regulating the gut microbiota and host health. A previous study showed that the intestinal microbiota of obese mice was significantly different from that of lean mice, indicating that obesity can affect the balance of intestinal microbiota [16]. In turn, obesity can be partially inhibited by improving intestinal microbiota. Studies in obese animal models also showed that gut microbiota can affect energy metabolism and energy storage by changing the production of SCFA or the secretion of intestinal cells [12,17,18]. In addition, the relative abundance of *Firmicutes* and *Bacteroidetes* in the intestinal microbiota is closely related to obesity, and their ratio is significantly positively related to animal obesity; furthermore, this feature is transmissible [19].

The occurrence and development of diabetes are closely related to the imbalance of intestinal microbiota, and the composition change in intestinal microbiota may cause probiotics to have an antidiabetes effect. Zhang et al. [20] reported that *Ruminococcus bromii* could reduce blood glucose by inhibiting the biotransformation of Deoxycholic acid. Furthermore, it has been shown that probiotics can significantly reduce the abundance of *Firmicutes* and increase the abundance of *Bacteroidetes* and GLP-1 and GLP-2 produced by enteroendocrine L cells to improve the glucose tolerance, leptin sensitivity, and metabolism level of mice [21], indicating that beneficial flora has an important influence on leptin signal transmission. In addition, in a mouse model, Cani et al. [22] showed that *Bifidobacterium species* can significantly improve diabetes.

Intestinal microbiota also has therapeutic effects on inflammation and diarrhea. Hung et al. [23] found that *Lactobacillus casei* can significantly inhibit intestinal inflammation in children to improve diarrhea. Yang et al. [24] found that *Lactobaccilus plantarum CCFM1143* can significantly reduce the abundance of *Bacteroides* and *Eggerthella* and enrich the abundance of *Akkermansia*, *Anaerostipes*, and *Terrisporobacter* in human intestinal microbiota, thereby inhibiting the increase in inflammatory factors such as IL-6, so as to improve inflammation and chronic diarrhea.

In humans, the number of pathogenic bacteria such as *Enterobacteria* and *Enterococcus faecalis* in the intestinal microbiota of the elderly is high, while the number of *Lactobacillus* is low, which may not only result in poor health but also lead to increased risk of infection [25]. It has also been shown that the mechanism of intestinal microbiota affecting bone mineral density may involve the immune system, which in turn regulates osteoblast production [26]. In addition, it was found that SCFAs can regulate inflammation and indirectly regulate osteoclast production by affecting T cells in the colon, thus affecting bone formation [27]. Thirdly, intestinal microbiota can inhibit the generation of osteoblasts and enhance the absorption and synthesis of various vitamins and minerals, including vitamins K and B12, calcium, and magnesium, which increase bone density and strength [28].

## 3. The Effect and Application Potential of Plant-Derived Bioactive Components on Gut Microflora

### 3.1. Effect of Curcumin on Gut Microflora

Curcumin, a bioactive substance originating from the rhizomes of Zingiberaceae and Araceae plants, is the main active ingredient in the spice curcuma. It has a long history of use as herbal medicine, dietary spice, and food colorant in East Asia. The dietary curcumin entering into the intestine is usually difficult to be directly absorbed, but it can be decomposed by intestinal microbiota after entering into the host, and affects health by changing the abundance of intestinal microbiota. Common curcumin-decomposing bacteria include *Escherichia*, *Blautia*, *Bifidobacterium*, *Lactobacillus*, *Pichia anomala*, *Bacillus megaterium dumb-002*, etc. [29]. Mounting evidence has shown that curcumin can not only significantly reduce the abundance of pathogenic bacteria such as *Prevotellaceae*, *Enterobacteria*, and *Rikenellaceae* in the intestinal microbiota, but also can significantly increase the abundance of beneficial bacteria such as *Bacteroides*, butyrate-producing bacteria, and *Lactobacillus*, so as to realize the biological functions of curcumin in regulating intestinal health [30,31]. In addition, in the dietary-induced obese mice, it was shown that curcumin can be metabolized into curcumin-O-glucuronide by the intestinal microbiota, and can significantly increase the relative abundance of *Lactococcus*, *Parasutterella,* and *Turicibacter* in the intestinal microbiota, so as to play an important role in reducing inflammation [32]. Li et al. [33] have demonstrated that dietary curcumin treatment (0.2%) in HFD-fed obese mice is able to change the intestinal microbiota composition by both reducing the proportion of *Firmicutes* and *Bacteroides,* as well as the abundance of endotoxin-producing *Desulfovibrio* bacteria, while increasing the abundance of SCFAs-producing bacteria such as *Akkermansia*, *Bacteroides*, *Parabacteroides*, *Alistipes,* and *Alloprevotella* in intestinal microbiota, which subsequently led to significantly promoted recovery of intestinal dysbiosis by increasing the concentration of SCFAs in the cecum and colon, and significantly reduced fat content, liver steatosis, lipopolysaccharide level, and insulin sensitivity. Zhang et al. [34] showed that supplementation of the metabolite dideoxycurcumin (150 mg/kg) in the diet of broilers could significantly increase the content of GSH, the activity of SOD and GR, and the content of GSSG in the jejunum, and increased the content of GSH, the activity of SOD and GST, and the redox potential in the ileum. These results show that curcumin is able to exert its protective effect on intestinal health by promoting the transformation of pathogenic bacteria to beneficial bacteria by reducing the number of harmful bacteria or increasing the abundance of beneficial bacteria in the intestine. Although curcumin is safe even at high doses in humans, it exhibits poor bioavailability. In human tests, it was found that curcumin could not be detected in the serum of subjects taking 500, 1000, 2000, 4000, 6000 or 8000 mg, and only low levels of curcumin could be detected in two subjects taking 10,000 or 12,000 mg [35]. In experiments using rats, even more than 90% of curcumin was excreted in the feces [36]. Major reasons contributing to the low plasma and tissue levels of curcumin appear to be due to poor absorption, rapid metabolism, and rapid systemic elimination [37].

### 3.2. Effect of Capsaicin on Gut Microflora

Peppers contain a variety of high-quality, plant-derived chemically active substances, including capsaicin, vitamin C, phenolic compounds, flavonoids, and carotenoids, which have significant antioxidant activity [38]. Capsaicin is the main bioactive component of red pepper (*Capsicum* L.), which has a variety of biological functions, such as lowering serum lipids, anti-inflammation, anticancer, antioxidation, and intestinal movement regulation. It has been demonstrated that capsaicin regulates intestinal function mainly by activating the expression of transient receptor potential vanillin 1 (TRPV1) [39]. Dietary capsaicin significantly increased the ratio of *Firmicutes*/*Bacteroidetes* and the abundance of *Roseburia flora*, and decreased the abundance of *Bacteroides* and *Parabacteroides* in intestinal microorganisms of mice, both low capsaicin and high capsaicin diets significantly increased the butyrate in feces and the total plasma GLP-1 level, compared with a normal diet; total plasma ghrelin and TNF-α, IL-1β, and IL-6 levels were all decreased. Dietary capsaicin helps to improve glucose homeostasis by increasing SCFA, regulate gastrointestinal hormones, and inhibit proinflammatory cytokines, thereby significantly improving the tolerance of mice to glucose and insulin, and significantly inhibiting the increase in fasting glucose and insulin levels [40]. In addition, Kang et al. [41] also found a similar conclusion in human studies, showing that dietary capsaicin (200–400 ppm) can reduce obesity and promote intestinal health by increasing the *Fractions*/*Bacteroidetes* ratio and *Faecalibacterium* abundance, and at the same time increasing the level of plasma glucagon such as GLP-1 and GIP. Hui et al. [42] further studied and found that capsaicin (100 μmol/L) reshapes intestinal microbiota, which is related to the production of LCA, an endogenous agonist of TGR5, which may enhance the secretion of GLP-1 in endogenous L cells. At the same time, fecal bacteria transplantation experiments showed that the beneficial effect of capsaicin induction is reversible, which indicates that the effect of capsaicin on glucose homeostasis depends largely on intestinal microbiota [42]. Moreover, dietary capsaicin can also inhibit the CB1 receptor and reduce the synthesis of LPS by increasing the abundance of butyric acid-producing bacteria such as *Ruminococcaceae and Lachnospiraceae* and butyrate level, thereby prevent metabolic endotoxemia and systemic chronic low-grade inflammation induced by HFD [43]. Kawada et al. [44] studied the absorption of capsaicin and dihydrocapsaicin (metabolites of capsaicins) in rats; the results showed that about 85% of the dose was absorbed by the gastrointestinal tract within 3 h. The absorption rates of capsaicin and dihydrocapsaicin in the stomach, jejunum, and ileum within 60 min were about 50%, 80%, and 70%, respectively. However, prolonged exposure to high doses of capsaicin (more than 100 mg/kg) led to peptic ulcer, accelerated the development of prostate, gastric, duodenal, and liver cancer, and enhanced the metastasis of breast cancer [45].

### 3.3. Effect of Quercetin on Gut Microflora

Quercetin is one of the most common flavonoids in nature, which is abundant in onions, kale, and apples, among other fruits and vegetables. Quercetin derived from different tissues has variable bioavailability. For example, compared with apple peel, quercetin derived from onion peel has higher bioavailability. Quercetin is one of the polyphenols that has been studied extensively at present and has a variety of physiological functions such as antibacterial, antioxidant, anti-inflammatory, and metabolic promotion [46]. Previous study has shown that quercetin treatment can significantly reduce the abundance of *Escherichia coli* and *Clostridium*, while significantly increasing the number of *Lactobacillus*, so as to improve the intestinal microbiota environment [47]. In addition, quercetin is also believed to significantly promote the intestinal barrier function of the human intestinal Caco-2 cell monolayer. Suzuki and Hara [48] have further reported that quercetin-enhanced (100 μmol/L) intestinal barrier function is contributed by an inhibited protein kinase pathway and a promoted relative expression of ZO-1, closure protein, and intercellular tight junction proteins, including occludin-3 and claudin-1. Using piglets, Xu et al. [49] have shown that dietary quercetin (0.1%) significantly decreased fecal scores, improved intestinal damage, and increased antioxidant capacity indices, by increasing the relative abundances of *Fibrobacteres*, *Akkermansia muciniphila*, *Clostridium butyricum*, *Clostridium celatum,* and *Prevotella copri*, and decreasing the relative abundances of *Proteobacteria*, *Lactobacillus coleohominis,* and *Ruminococcus bromii*, suggesting that dietary quercetin supplementation attenuated diarrhea and intestinal damage by enhancing the antioxidant capacity and regulating gut microbial structure and metabolism. Amasheh et al. [50] comprehensively evaluated the effect of quercetin on the expression of cytokines in rat colon and the intestinal barrier damage induced by TNF-α and IFN-γ, and found that quercetin can effectively protect the intestinal barrier function by downregulating claudin-2 protein abundance. The permeability analysis of rat colon in vitro showed that quercetin can reduce the total resistance of the intestinal barrier by partially inhibiting the cytokine TNF-α and IFN-γ. Studies have also shown that quercetin can regulate the abundance of the intestinal microbiota of *Adlercreutzia*, *Allobaculum*, *Coprococcus_1*, *Lactococcus*, and *Akkermansia*, so as to improve HFD-induced intestinal microbiota-related diseases, and significantly alleviate HFD-induced obesity, improve glucose tolerance, restore intestinal barrier function, and reduce adipose tissue inflammation [51]. Chen et al. [52] found that the bioavailability of quercetin was very low and only 5.3% of the constant quercetin had bioavailability. After oral administration, although the total amount of quercetin absorbed was about 59.1%, and about 93.3% of quercetin was metabolized in the intestine, only 3.1% was metabolized in the liver, and nonsignificant enterohepatic recirculation was observed for quercetin and its conjugated metabolites.

### 3.4. Effect of Resveratrol on Gut Microflora

Resveratrol is a natural polyphenol compound that is widely found in grapes, *Polygonum cuspidatum*, peanuts, and other plants [53], and has a variety of biological effects, including anti-inflammatory [54], antioxidant stress [55], and regulation of energy metabolism [56]. Zhao et al. [57] reported that resveratrol can maintain the integrity of the intestinal barrier and reduce intestinal damage by inhibiting the apoptosis of intestinal epithelial cells in rats. Furthermore, it has been demonstrated that resveratrol can regulate the composition of intestinal microbiota and reduce the number of opportunistic pathogens in the body. Yang et al. [58] showed that, compared with the control group, the intestinal microbiota diversity of HFD-fed rats was significantly reduced and the level of oxidative stress was increased. It has also been shown that resveratrol supplementation (400 mg/kg) can significantly increase the number of butyric acid-producing bacteria such as *Blautia* and *Dorea* in the intestinal microbiota. In addition, dietary resveratrol is able to significantly reduce the number of inflammation-related bacteria such as *Bacteroides* and *Desulfovibrionaceaesp*, most of which are antibiotic-resistant bacteria and may become highly pathogenic bacteria. Pan et al. [59] also found that resveratrol treatment (10%) can significantly inhibit the flora abundance of *Desulfovibrio* and *Lachnospiraceae*_NK4A136_group in the intestine, which significantly reduces the enrichment of pathways related to host metabolic diseases, and increases the enrichment of pathways involved in the production of small metabolites. In addition, 4-HPA and 3-HPP are two intestinal metabolites of resveratrol, which help to improve lipid metabolism in vitro. Qiao et al. [60] have proved that in HFD-fed mice, resveratrol (400 mg/kg) supplementation can increase the ratio of *Bacteroides* and *Firmicutes*, together with the number of *Lactobacillus* and *Bifidobacterium*, while significantly reduce the abundance of *Enterococcus faecalis* in the intestinal microbiota. Previous study in mice has also shown that resveratrol supplementation can increase the relative abundance of *Lactobacillus* and *Bifidobacterium* in the intestine, thus increasing the bile acid synthesis in the liver to ameliorate atherosclerosis induced by trimethylamine-N-oxide (TMAO) [61]. Additionally, further research showed that resveratrol was able to protect the host from colitis by reversing the development of pathogenic microbiota such as *Bacteroides acidifaciens* and the decline of beneficial bacteria such as *Rinococcus gnavus* and *Akkermansia mucinphilia* to inhibit inflammatory Th1/Th17 cells [62]. The oral absorption of resveratrol by human beings is about 75%. The extensive metabolism in the intestine and liver leads to the oral bioavailability being significantly less than 1%, and the increase in resveratrol dosage and repeated administration do not seem to significantly change this [63].

### 3.5. Effect of Allicin on Gut Microflora

Garlic contains numerous bioactive compounds, such as allicin, organic sulfur, flavonoids, saponins, phenols, and polysaccharides. Using C57BL/6N male obese mice as models, Chen et al. [64] explored the effect of whole garlic (1%) on intestinal microbiota and the results showed that treatment with whole garlic for 12 weeks could significantly reduce the effect of HFD on GPT/GOT and TCHO, LDL-c levels, and HOMA-IR. Furthermore, the addition of whole garlic significantly improved the ratio of intestinal villus height to crypt depth, the weight of caecum, and the concentration of caecal organic acid decreased by HFD. Finally, the 16S rRNA sequencing results of intestinal microorganisms showed that the addition of garlic significantly increased the α-diversity and the abundance of *Lachnospiraceae*, while decreased the abundance of *Prevotella*. Wu et al. [65] have showed that after oral administration of melanoidin from black garlic, the intestinal microbiota of HFD-induced obese mice was significantly improved, and the bacterial diversity and richness were significantly increased. Furthermore, allicin (200 mg/kg) can significantly improve HFD-induced obesity in mice by increasing the abundance of *Bacteroidaceae* and other probiotics such as *Lactobacilliceae* and *Akkermansiaceae* in the intestinal microbiota and reducing the abundance of *Enterobacteriaceae* and *Desulfovibrionaceae*. In addition, both in vitro and in vivo studies have shown that raw garlic juice and allicin can significantly inhibit the production of human intestinal flora γBB and TMA and result in a significant reduction in the production of TMAO, indicating that both raw garlic juice and allicin can reduce the risk of human cardiovascular diseases by regulating intestinal microbiota [66]. In a rat model of Acrylamide-induced intestinal injury, allicin treatment significantly reversed the reduction in SCFA synthesis bacteria such as *Escherichia_Shigella*, *Dubosiella*, and *Alloprevotella*, together with the reduction in acetic acid and propionic acid. Furthermore, allicin significantly increased the expression of occludin, claudin-1, ZO-1, mucin 2, and mucin 3, and significantly decreased the expression of TLR4, MyD88, NF-κB signaling pathway proteins, and proinflammatory cytokines. These results show that allicin was able to significantly improve intestinal mucosal barrier damage and inflammation induced by Acrylamide [67].

### 3.6. Effect of Catechin on Gut Microflora

Catechins are a kind of flavonoid mainly derived from green tea, which have high biological activities and play a key regulatory role in the antioxidation, antimutagenesis, anticancer, anticardiovascular diseases, antibacterial, anti-inflammatory and weight loss. Catechins prepared from 2 g of dry leaves and 100 mL of boiling distilled water can inhibit the growth rate of pathogenic bacteria such as *H. pylori*, *Staphylococcus aureus*, *E. coli O157: H7*, *Salmonellatyphimurium DT104*, *Pseudomonas aeruginosa* in the intestinal microbiota [68]. Additionally, Liao et al. [69] have found that catechin (0.4–1.6 g/L) was able to increase the number of *Bifidobacterium* in the intestinal tract of mice, and could reduce the levels of TCHO and LDL-c. Studies have shown that after ingestion by the body, catechin can be decomposed into small molecular substances under the metabolism of colonic microbiota to enter into hepatointestinal circulation or blood circulation, playing a wide range of physiological functions [70]. EGCG and GCG, the main components of catechin, can also significantly reduce the abundance of harmful bacteria such as *Bacteroides*, *Prevotella*, *Clostridium histolyticum*, *Eubacterium,* and *Clostridium* [71]. Liu et al. [72] have also demonstrated that EGCG can significantly promote the growth of probiotics such as *Bacteroides*, *Christensenellaceae*, and *Bifidobacterium* in the human intestine through secondary metabolites, such as 4-phenylbutyric acid and phenylacetic acid, and significantly inhibit the growth of *Fusobacterium varium*, *Bilophila,* and *Enterobacteriaceae*. Studies have also shown that EGCG can significantly increase the *Firmicutes*/*Bacteroidetes* ratio and the abundances of the *Lactobacillus gasseri*, *Lactobacillus intestinalis*, *Christensenellaceae*, and *Lactobacillus reuteri*, and decreased the abundances of the *Enterobacteriaceae* and *Proteobacteria* to improve glucose homeostasis in diabetic mice by decreasing serum cholesterol and LDL levels and increasing the HDL/LDL ratio [73]. These results indicate that catechins and their metabolites are able to promote the health of host animals by changing the abundance of specific intestinal microbiota. However, the bioavailability of catechins is very low. Previous studies showed that the oral bioavailability of EGCG in rats is only about 4.9% [74]. In human studies, ingested catechin and its metabolites reach a peak in plasma after 5 h, and only about 1.68% of the ingested catechins are present in plasma, urine, and feces [75].

### 3.7. Effect of Lignans on Gut Microflora

Lignans are complex diphenolic compounds representing phytoestrogens and occur widely across the plant kingdom, having estrogen and antiestrogen characteristics [76]. Lignans are widely found in natural foods. Wheat and rye bran have the highest lignan content in all cereals and among the species studied so far; flaxseed and sesame are the most abundant substances containing lignan [77]. Previous research has shown that the intestinal bacteria, *Clostridium saccharogumia*, *Eggerthella lenta*, *Blautia producta,* and *Lactonifactor longoviformis*, were able to convert lignan into bioactive enterolignans enterodiol, and enterolactone [78], and thereby further regulate cholesterol [79], reduce cardiovascular disease [80], and play the role of anti-inflammation and antioxidation [81] agents in regulating health. Further studies have shown that dietary lignans play an anti-inflammatory role by targeting NF-κB [82]. Li et al. [83] found that 3633 μg/day of total lignans were able to significantly enhance metabolic health, and found that the abundance of *Coprococcus* sp. ART55/1, *Faecalibacterium prausnitzii*, *Alistipes shahii*, *Butyrivibrio crossotus*, and *Methanobrevibacter smithii* had a significantly positive correlation with the level of enterolactone, while the abundance of *Bacteroides dorei*, *Bacteroides fragilis*, *Clostridium bolteae*, *Clostridium leptum*, *Clostridium symbiosum*, *Lachnospiraceae bacterium*. 1.4.56FAA, and *Ruminococcus* sp. 5.1.39BFAA was negatively correlated with the level of enterolactone. In a study on rats, Xiao et al. [84] showed that lignans mainly escalated the abundance of *Antinobacteria* and modulated several genera relating to bone mineral density, achieving the effect of alleviated bone loss and decreased serum levels of serotonin.

**Figure 2:**
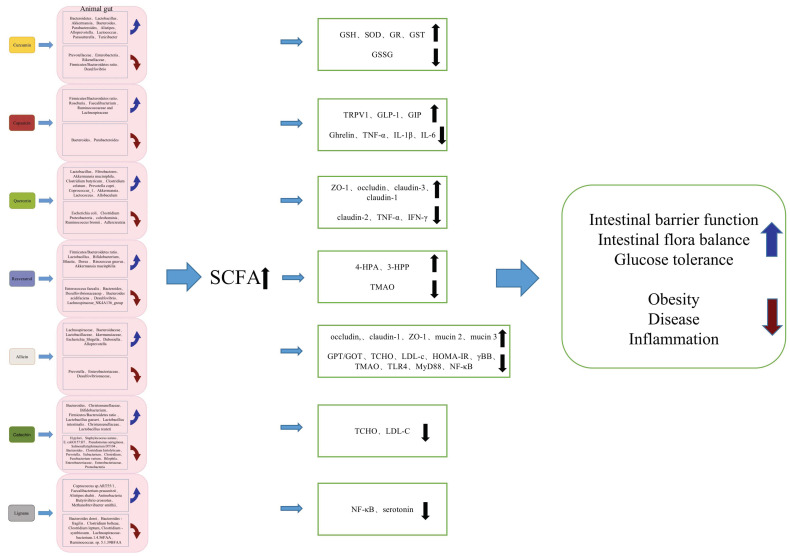
Functions of plant-derived bioactive compounds in metabolic disorders by regulating intestinal SCFA production.

Through these studies investigating the gut microbiome and its relevance with metabolic disorders, it can be concluded that plant-derived bioactive compounds, such as curcumin, capsaicin, allicin, quercetin, resveratrol, catechin, and lignans play an important role in the regulation of multiple aspects of metabolic disorders, by regulating the composition of gut microflora. The interactions between gut microbiota and plant-derived bioactive compounds are mainly achieved by the following two pathways: one is the modulation of the gut microbiota profile by ingested natural compounds, and the other is the gut microbial conversion of natural products into ”daughter molecules” with potent bioactivities (Table 1). Furthermore, the bacterial metabolites involved in these interactions are very diverse and range from small molecules to large macromolecules, including byproducts of bacterial metabolisms such as SCFAs, and various gut microbes have already been extensively documented to contribute to the metabolization of these nondigestible carbohydrates into SCFAs (Figure 2).

## 4. Conclusions

**Figure 3:**
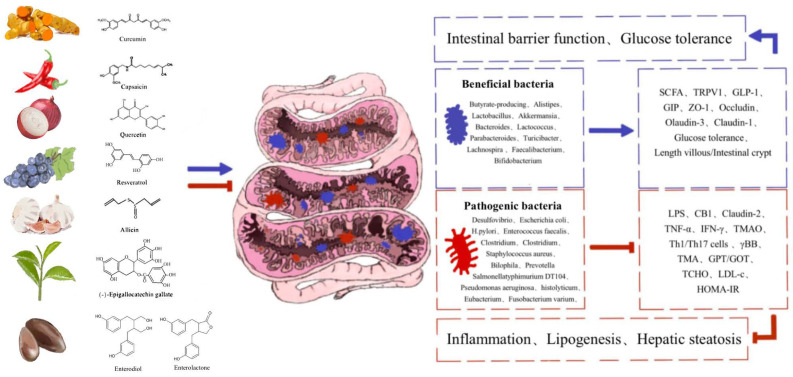
Plant-derived bioactive compounds improve host health by modulating the intestinal microbiota balance.

Taken together, intestinal microbiota plays an important role in improving host health, and the plant-derived bioactive compounds have a positive effect on the health (the intestine and the whole body) of host animals by increasing the abundance of beneficial bacteria and reducing the abundance of harmful bacteria, helping to improve intestinal microbial balance, metabolism, and intestinal integrity (Figure 3). As a potential substitute for antibiotics, plant-derived bioactive compounds have gradually shown great potential for application in the fields of biomedicine and animal husbandry and veterinary medicine, by regulating various biological processes of the animal organism through changing the composition and abundance of intestinal microbiota. However, the low blood concentration, low bioavailability and strong toxicity of plant-derived active ingredients have greatly reduced their medicinal potential, thus limiting their clinical application prospects. Therefore, on one hand, it is urgent to further explore reliable methods to improve their bioavailability (such as preparation of micelles, liposomes, phospholipid complexes, microemulsions, nanostructured lipid carriers, and biopolymer nanoparticles), and on the other hand, it is also of great importance to further explore the potential biological functions of plant-derived active ingredients, especially the potential effects of interactions with intestinal flora in maintaining body health and disease treatment, so as to promote their wide application in various fields. In addition, it is also necessary to further determine the potential metabolic mechanism of intestinal microbiota and the impact of metabolites generated from plant-derived active ingredients after the transformation of intestinal microbiota on the intestinal microbiota composition. This would facilitate the application of plant-derived active ingredients in biological medicine, animal husbandry, and veterinary medicine, making a large contribution to the sustainable and healthy development of humans and modern animal husbandry.

## Figures and Tables

**Table 1:** Interactions between metabolites of gut microflora and plant-derived bioactive compounds.

Plant-Derived Bioactive Compounds	Main Active Ingredients	Metabolic Gut Microflora	Reference
Curcumin	Curcumin-O-glucuronide	*Lactococcus* *Parasutterella* *Turicibacter*	[32]
Quercetin	3,4-Dihydroxyphenylacetic acid	*B. fragilis*, *C. perfringens*, *E. ramulus*, *Streptococcus S-2*, *Lactobacillus L-2*, *Bifidobacterium B-9* *Bacteroides JY-6*	[85]
Resveratrol	Dihydroresveratrol 3,4′-Dihydroxy-trans-stilbene 3,4′-Dihydroxybibenzyl	*Slackia equolifaciens* *Adlercreutzia equolifacens*	[86]
Catechin	Valerolactones Hydroxyvaleric acid	*Eggertella lenta* *Flavonifractor plautii*	[85]
Lignans	Enterolignans enterodiol Enterolactone	*Clostridium saccharogumia Eggerthella lenta* *Blautia producta Lactonifactor longoviformis*	[78]

## Data Availability

Not applicable.

## Abbreviations

SCFA	short-chain fatty acids
ND	normal diet
HFD	high fat diet
TRPV1	transient receptor potential vanilloid 1
GLP-1	glucagon-like peptide-1
GIP	gastric inhibitory polypeptide
CB1	cannabinoid receptor 1
LPS	lipopolysaccharide
ZO-1	zonula occludens 1
TNF-α	tumor necrosis factor-alpha
TNF-γ	tumor necrosis factor-gamma
TMAO	trimetlylamine oxide
Th1	helper T cell 1
Th17	helper T cell 17
GPT	glutamic-pyruvic transaminase
GOT	glutamic oxaloacetic transa minase
TCHO	total cholesterol
LDL-c	low-density lipoprotein cholesterol
GSH	glutathione
SOD	superoxide dismutase
GR	glutathione reductase
GST	glutathione S-transferase
GSSG	oxidized glutathione
LCA	lithocholic acid
TGR5	takeda G-protein coupled receptor 5
4-HPA	4-hydroxyphenylacetic acid
3-HPP	3-hydroxyphenylpropionic acid
TLR4	toll-like receptor 4
MyD88	myeloid differentiation factor 88
LDL	low-density lipoprotein
HOMA-IR	homeostatic model assessment for insulin resistance
γBB	γ-butyrobetaine
TMA	trimethylamine
EGCG	epigallocatechin gallate
GCG	gallocatechin galleate

## References

[1] Anomaly J. (2009). Harm to Others: The Social Cost of Antibiotics in Agriculture. J. Agric. Environ. Ethics.

[2] Lange K., Buerger M., Stallmach A., Bruns T. (2016). Effects of Antibiotics on Gut Microbiota. Dig. Dis..

[3] Bäckhed F., Ley R.E., Sonnenburg J.L., Peterson D.A., Gordon J.I. (2005). Host-Bacterial Mutualism in the Human Intestine. Science.

[4] Berg R. (1996). The indigenous gastrointestinal microflora. Trends Microbiol..

[5] Sittipo P., Shim J., Lee Y. (2019). Microbial Metabolites Determine Host Health and the Status of Some Diseases. Int. J. Mol. Sci..

[6] Mirzaei R., Bouzari B., Hosseini-Fard S.R., Mazaheri M., Ahmadyousefi Y., Abdi M., Jalalifar S., Karimitabar Z., Teimoori A., Keyvani H. (2021). Role of microbiota-derived short-chain fatty acids in nervous system disorders. Biomed. Pharmacother..

[7] Louis P., Flint H.J. (2009). Diversity, metabolism and microbial ecology of butyrate-producing bacteria from the human large intestine. FEMS Microbiol. Lett..

[8] Louis P., Flint H.J. (2017). Formation of propionate and butyrate by the human colonic microbiota. Environ. Microbiol..

[9] Rivière A., Selak M., Lantin D., Leroy F., De Vuyst L. (2016). Bifidobacteria and Butyrate-Producing Colon Bacteria: Importance and Strategies for Their Stimulation in the Human Gut. Front. Microbiol..

[10] Derrien M., Vaughan E.E., Plugge C.M., de Vos W.M. (2004). Akkermansia muciniphila gen. nov., sp. nov., a human intestinal mucin-degrading bacterium. Int. J. Syst. Evol. Microbiol..

[11] Mahowald M.A., Rey F.E., Seedorf H., Turnbaugh P.J., Fulton R.S., Wollam A., Shah N., Wang C., Magrini V., Wilson R.K. (2009). Characterizing a model human gut microbiota composed of members of its two dominant bacterial phyla. Proc. Natl. Acad. Sci. USA.

[12] Heimann E., Nyman M., Degerman E. (2015). Propionic acid and butyric acid inhibit lipolysis and de novo lipogenesis and increase insulin-stimulated glucose uptake in primary rat adipocytes. Adipocyte.

[13] Kelly C.J., Zheng L., Campbell E.L., Saeedi B., Scholz C.C., Bayless A.J., Wilson K.E., Glover L.E., Kominsky D.J., Magnuson A. (2015). Crosstalk between Microbiota-Derived Short-Chain Fatty Acids and Intestinal Epithelial HIF Augments Tissue Barrier Function. Cell Host Microbe..

[14] Arpaia N., Campbell C., Fan X., Dikiy S., van der Veeken J., deRoos P., Liu H., Cross J.R., Pfeffer K., Coffer P.J. (2013). Metabolites produced by commensal bacteria promote peripheral regulatory T-cell generation. Nature.

[15] Krüger M., Neuhaus J., Herrenthey A.G., Gökce M.M., Schrödl W., Shehata A.A. (2014). Chronic botulism in a Saxony dairy farm: Sources, predisposing factors, development of the disease and treatment possibilities. Anaerobe.

[16] Ley R.E., Bäckhed F., Turnbaugh P., Lozupone C.A., Knight R.D., Gordon J.I. (2005). Obesity alters gut microbial ecology. Proc. Natl. Acad. Sci. USA.

[17] Chimerel C., Emery E., Summers D.K., Keyser U., Gribble F.M., Reimann F. (2014). Bacterial metabolite indole modulates incretin secretion from intestinal enteroendocrine L cells. Cell Rep..

[18] Bäckhed F., Ding H., Wang T., Hooper L.V., Koh G.Y., Nagy A., Semenkovich C.F., Gordon J.I. (2004). The gut microbiota as an environmental factor that regulates fat storage. Proc. Natl. Acad. Sci. USA.

[19] Turnbaugh P.J., Ley R.E., Mahowald M.A., Magrini V., Mardis E.R., Gordon J.I. (2006). An obesity-associated gut microbiome with increased capacity for energy harvest. Nature.

[20] Zhang Y., Gu Y., Ren H., Wang S., Zhong H., Zhao X., Ma J., Gu X., Xue Y., Huang S. (2020). Gut microbiome-related effects of berberine and probiotics on type 2 diabetes (the PREMOTE study). Nat. Commun..

[21] Everard A., Lazarevic V., Derrien M., Girard M., Muccioli G.G., Neyrinck A.M., Possemiers S., Van Holle A., François P., de Vos W.M. (2011). Responses of gut microbiota and glucose and lipid metabolism to prebiotics in genetic obese and diet-induced leptin-resistant mice. Diabetes.

[22] Cani P.D., Neyrinck A.M., Fava F., Knauf C., Burcelin R.G., Tuohy K.M., Gibson G.R., Delzenne N.M. (2007). Selective increases of bifidobacteria in gut microflora improve high-fat-diet-induced diabetes in mice through a mechanism associated with endotoxaemia. Diabetologia.

[23] Lai H.-H., Chiu C.-H., Kong M.-S., Chang C.-J., Chen C.-C. (2019). Probiotic Lactobacillus casei: Effective for Managing Childhood Diarrhea by Altering Gut Microbiota and Attenuating Fecal Inflammatory Markers. Nutrients.

[24] Yang B., Yue Y., Chen Y., Ding M., Li B., Wang L., Wang Q., Stanton C., Ross R.P., Zhao J. (2021). Lactobacillus plantarum CCFM1143 Alleviates Chronic Diarrhea via Inflammation Regulation and Gut Microbiota Modulation: A Double-Blind, Randomized, Placebo-Controlled Study. Front. Immunol..

[25] Van Tongeren S.P., Slaets J.P.J., Harmsen H.J.M., Welling G.W. (2005). Fecal Microbiota Composition and Frailty. Appl. Environ. Microbiol..

[26] Ohlsson C., Sjögren K. (2015). Effects of the gut microbiota on bone mass. Trends Endocrinol. Metab..

[27] Charles J.F., Ermann J., Aliprantis A.O. (2015). The intestinal microbiome and skeletal fitness: Connecting bugs and bones. Clin. Immunol..

[28] Inglis J.E., Ilich J.Z. (2015). The Microbiome and Osteosarcopenic Obesity in Older Individuals in Long-Term Care Facilities. Curr. Osteoporos. Rep..

[29] Shen L., Ji H.-F. (2019). Bidirectional interactions between dietary curcumin and gut microbiota. Crit. Rev. Food Sci. Nutr..

[30] Shen L., Liu L., Ji H.-F. (2017). Regulative effects of curcumin spice administration on gut microbiota and its pharmacological implications. Food Nutr. Res..

[31] Sun Z.-Z., Li X.-Y., Wang S., Shen L., Ji H.-F. (2020). Bidirectional interactions between curcumin and gut microbiota in transgenic mice with Alzheimer’s disease. Appl. Microbiol. Biotechnol..

[32] Islam T., Koboziev I., Albracht-Schulte K., Mistretta B., Scoggin S., Yosofvand M., Moussa H., Zabet-Moghaddam M., Ramalingam L., Gunaratne P.H. (2021). Curcumin Reduces Adipose Tissue Inflammation and Alters Gut Microbiota in Diet-Induced Obese Male Mice. Mol. Nutr. Food Res..

[33] Li S., You J., Wang Z., Liu Y., Wang B., Du M., Zou T. (2021). Curcumin alleviates high-fat diet-induced hepatic steatosis and obesity in association with modulation of gut microbiota in mice. Food Res. Int. Ott. Ont.

[34] Zhang J., Han H., Zhang L., Wang T. (2021). Dietary bisdemethoxycurcumin supplementation attenuates lipopolysaccharide-induced damages on intestinal redox potential and redox status of broilers. Poult. Sci..

[35] Lao C.D., Ruffin M.T., Normolle D., Heath D.D., Murray S.I., Bailey J.M., Boggs M.E., Crowell J., Rock C.L., Brenner D.E. (2006). Dose escalation of a curcuminoid formulation. BMC Complement. Altern. Med..

[36] Metzler M., Pfeiffer E., Schulz S.I., Dempe J.S. (2013). Curcumin uptake and metabolism. BioFactors.

[37] Anand P., Kunnumakkara A.B., Newman R.A., Aggarwal B.B. (2007). Bioavailability of curcumin: Problems and promises. Mol. Pharm..

[38] Alvarez-Parrilla E., de la Rosa L.A., Amarowicz R., Shahidi F. (2011). Antioxidant Activity of Fresh and Processed Jalapeño and Serrano Peppers. J. Agric. Food Chem..

[39] Holzer P. (2004). TRPV1 and the gut: From a tasty receptor for a painful vanilloid to a key player in hyperalgesia. Eur. J. Pharmacol..

[40] Song J.-X., Ren H., Gao Y.-F., Lee C.-Y., Li S.-F., Zhang F., Li L., Chen H. (2017). Dietary Capsaicin Improves Glucose Homeostasis and Alters the Gut Microbiota in Obese Diabetic ob/ob Mice. Front. Physiol..

[41] Kang C., Zhang Y., Zhu X., Liu K., Wang X., Chen M., Wang J., Chen H., Hui S., Huang L. (2016). Healthy Subjects Differentially Respond to Dietary Capsaicin Correlating with Specific Gut Enterotypes. J. Clin. Endocrinol. Metab..

[42] Hui S., Huang L., Wang X., Zhu X., Zhou M., Chen M., Yi L., Mi M. (2020). Capsaicin improves glucose homeostasis by enhancing glucagon-like peptide-1 secretion through the regulation of bile acid metabolism via the remodeling of the gut microbiota in male mice. FASEB J..

[43] Kang C., Wang B., Kaliannan K., Wang X., Lang H., Hui S., Huang L., Zhang Y., Zhou M., Chen M. (2017). Gut Microbiota Mediates the Protective Effects of Dietary Capsaicin against Chronic Low-Grade Inflammation and Associated Obesity Induced by High-Fat Diet. mBio.

[44] Kawada T., Suzuki T., Takahashi M., Iwai K. (1984). Gastrointestinal absorption and metabolism of capsaicin and dihydrocapsaicin in rats. Toxicol. Appl. Pharmacol..

[45] Rollyson W.D., Stover C.A., Brown K.C., Perry H.E., Stevenson C.D., McNees C.A., Ball J.G., Valentovic M.A., Dasgupta P. (2014). Bioavailability of capsaicin and its implications for drug delivery. J. Control. Release Off. J. Control. Release Soc..

[46] Erlund I. (2004). Review of the flavonoids quercetin, hesperetin, and naringenin. Dietary sources, bioactivities, bioavailability, and epidemiology. Nutr. Res..

[47] Abdel-Latif M.A., Elbestawy A.R., El-Far A.H., Noreldin A.E., Emam M., Baty R.S., Albadrani G.M., Abdel-Daim M.M., Abd El-Hamid H.S. (2021). Quercetin Dietary Supplementation Advances Growth Performance, Gut Microbiota, and Intestinal mRNA Expression Genes in Broiler Chickens. Animals.

[48] Suzuki T., Hara H. (2009). Quercetin Enhances Intestinal Barrier Function through the Assembly of Zonnula Occludens-2, Occludin, and Claudin-1 and the Expression of Claudin-4 in Caco-2 Cells. J. Nutr..

[49] Xu B., Qin W., Xu Y., Yang W., Chen Y., Huang J., Zhao J., Ma L. (2021). Dietary Quercetin Supplementation Attenuates Diarrhea and Intestinal Damage by Regulating Gut Microbiota in Weanling Piglets. Oxid. Med. Cell. Longev..

[50] Amasheh M., Grotjohann I., Amasheh S., Fromm A., Söderholm J.D., Zeitz M., Fromm M., Schulzke J.-D. (2009). Regulation of mucosal structure and barrier function in rat colon exposed to tumor necrosis factor alpha and interferon gamma in vitro: A novel model for studying the pathomechanisms of inflammatory bowel disease cytokines. Scand. J. Gastroenterol..

[51] Su L., Zeng Y., Li G., Chen J., Chen X. (2022). Quercetin improves high-fat diet-induced obesity by modulating gut microbiota and metabolites in C57BL/6J mice. Phytother. Res. PTR.

[52] Chen X., Yin O.Q.P., Zuo Z., Chow M.S.S. (2005). Pharmacokinetics and modeling of quercetin and metabolites. Pharm. Res..

[53] Zhang C., Zhao X.H., Yang L., Chen X.Y., Jiang R.S., Jin S.H., Geng Z.Y. (2014). Resveratrol and cardiovascular health--promising therapeutic or hopeless illusion?. Pharmacol Res..

[54] Manna S.K., Mukhopadhyay A., Aggarwal B.B. (2000). Resveratrol Suppresses TNF-Induced Activation of Nuclear Transcription Factors NF-κB, Activator Protein-1, and Apoptosis: Potential Role of Reactive Oxygen Intermediates and Lipid Peroxidation. J. Immunol..

[55] Liu L.L., He J.H., Xie H.B., Yang Y.S., Li J.C., Zou Y. (2014). Resveratrol induces antioxidant and heat shock protein mRNA expression in response to heat stress in black-boned chickens. Poult. Sci..

[56] Lagouge M., Argmann C., Gerhart-Hines Z., Meziane H., Lerin C., Daussin F., Messadeq N., Milne J., Lambert P., Elliott P. (2006). Resveratrol Improves Mitochondrial Function and Protects against Metabolic Disease by Activating SIRT1 and PGC-1α. Cell.

[57] Zhao W., Huang X., Han X., Hu D., Hu X., Li Y., Huang P., Yao W. (2018). Resveratrol Suppresses Gut-Derived NLRP3 Inflammasome Partly through Stabilizing Mast Cells in a Rat Model. Mediat. Inflamm..

[58] Yang C., Deng Q., Xu J., Wang X., Hu C., Tang H. (2019). Sinapic acid and resveratrol alleviate oxidative stress with modulation of gut microbiota in high-fat diet-fed rats. Food Res. Int..

[59] Wang P., Gao J., Ke W., Wang J., Li D., Liu R., Jia Y., Wang X., Chen X., Chen F. (2020). Resveratrol reduces obesity in high-fat diet-fed mice via modulating the composition and metabolic function of the gut microbiota. Free Radic. Biol. Med..

[60] Qiao Y., Sun J., Xia S., Tang X., Shi Y., Le G. (2014). Effects of resveratrol on gut microbiota and fat storage in a mouse model with high-fat-induced obesity. Food Funct..

[61] Chen M., Yi L., Zhang Y., Zhou X., Ran L., Yang J., Zhu J., Zhang Q., Mi M. (2016). Resveratrol Attenuates Trimethylamine-N-Oxide (TMAO)-Induced Atherosclerosis by Regulating TMAO Synthesis and Bile Acid Metabolism via Remodeling of the Gut Microbiota. MBio.

[62] Alrafas H.R., Busbee P.B., Nagarkatti M., Nagarkatti P.S. (2019). Resveratrol modulates the gut microbiota to prevent murine colitis development through induction of Tregs and suppression of Th17 cells. J. Leukoc. Biol..

[63] Walle T. (2011). Bioavailability of resveratrol. Ann. N. Y. Acad. Sci..

[64] Chen K., Xie K., Liu Z., Nakasone Y., Sakao K., Hossain M.A., Hou D.-X. (2019). Preventive Effects and Mechanisms of Garlic on Dyslipidemia and Gut Microbiome Dysbiosis. Nutrients.

[65] Wu J., Liu Y., Dou Z., Wu T., Liu R., Sui W., Jin Y., Zhang M. (2020). Black garlic melanoidins prevent obesity, reduce serum LPS levels and modulate the gut microbiota composition in high-fat diet-induced obese C57BL/6J mice. Food Funct..

[66] Panyod S., Wu W.-K., Chen P.-C., Chong K.-V., Yang Y.-T., Chuang H.-L., Chen C.-C., Chen R.-A., Liu P.-Y., Chung C.-H. (2022). Atherosclerosis amelioration by allicin in raw garlic through gut microbiota and trimethylamine-N-oxide modulation. NPJ Biofilms Microbiomes.

[67] Yuan Y., Lu L., Bo N., Chaoyue Y., Haiyang Y. (2021). Allicin Ameliorates Intestinal Barrier Damage via Microbiota-Regulated Short-Chain Fatty Acids-TLR4/MyD88/NF-κB Cascade Response in Acrylamide-Induced Rats. J. Agric. Food Chem..

[68] Bancirova M. (2010). Comparison of the antioxidant capacity and the antimicrobial activity of black and green tea. Food Res. Int..

[69] Liao Z.-L., Zeng B.-H., Wang W., Li G.-H., Wu F., Wang L., Zhong Q.-P., Wei H., Fang X. (2016). Impact of the Consumption of Tea Polyphenols on Early Atherosclerotic Lesion Formation and Intestinal Bifidobacteria in High-Fat-Fed ApoE^−/−^ Mice. Front. Nutr..

[70] Pastoriza S., Mesías M., Cabrera C., Rufián-Henares J.A. (2017). Healthy properties of green and white teas: An update. Food Funct..

[71] Zhang X., Zhu X., Sun Y., Hu B., Sun Y., Jabbar S., Zeng X. (2013). Fermentation in vitro of EGCG, GCG and EGCG3”Me isolated from Oolong tea by human intestinal microbiota. Food Res. Int..

[72] Liu Z., de Bruijn W.J.C., Bruins M.E., Vincken J.-P. (2020). Reciprocal Interactions between Epigallocatechin-3-gallate (EGCG) and Human Gut Microbiota In Vitro. J. Agric. Food Chem..

[73] Park J.-M., Shin Y., Kim S.H., Jin M., Choi J.J. (2020). Dietary Epigallocatechin-3-Gallate Alters the Gut Microbiota of Obese Diabetic db/db Mice: Lactobacillus Is a Putative Target. J. Med. Food.

[74] Lin L.-C., Wang M.-N., Tseng T.-Y., Sung J.-S., Tsai T.-H. (2007). Pharmacokinetics of (−)-Epigallocatechin-3-gallate in Conscious and Freely Moving Rats and Its Brain Regional Distribution. J. Agric. Food Chem..

[75] Warden B.A., Smith L.S., Beecher G.R., Balentine D.A., Clevidence B.A. (2001). Catechins are bioavailable in men and women drinking black tea throughout the day. J. Nutr..

[76] Tham D.M., Gardner C.D., Haskell W.L. (1998). Clinical review 97: Potential health benefits of dietary phytoestrogens: A review of the clinical, epidemiological, and mechanistic evidence. J. Clin. Endocrinol. Metab..

[77] Smeds A.I., Eklund P.C., Sjöholm R.E., Willför S.M., Nishibe S., Deyama T., Holmbom B.R. (2007). Quantification of a broad spectrum of lignans in cereals, oilseeds, and nuts. J. Agric. Food Chem..

[78] Woting A., Clavel T., Loh G., Blaut M. (2010). Bacterial transformation of dietary lignans in gnotobiotic rats. FEMS Microbiol. Ecol..

[79] Creus-Cuadros A., Tresserra-Rimbau A., Quifer-Rada P., Martínez-González M.A., Corella D., Salas-Salvadó J., Fitó M., Estruch R., Gómez-Gracia E., Lapetra J. (2017). Associations between Both Lignan and Yogurt Consumption and Cardiovascular Risk Parameters in an Elderly Population: Observations from a Cross-Sectional Approach in the PREDIMED Study. J. Acad. Nutr. Diet..

[80] Witkowska A.M., Waśkiewicz A., Zujko M.E., Szcześniewska D., Stepaniak U., Pająk A., Drygas W. (2018). Are Total and Individual Dietary Lignans Related to Cardiovascular Disease and Its Risk Factors in Postmenopausal Women? A Nationwide Study. Nutrients.

[81] Soleymani S., Habtemariam S., Rahimi R., Nabavi S.M. (2020). The what and who of dietary lignans in human health: Special focus on prooxidant and antioxidant effects. Trends Food Sci. Technol..

[82] Li D., Luo F., Guo T., Han S., Wang H., Lin Q. (2022). Targeting NF-κB pathway by dietary lignans in inflammation: Expanding roles of gut microbiota and metabolites. Crit. Rev. Food Sci. Nutr..

[83] Li Y., Wang F., Li J., Ivey K.L., Wilkinson J.E., Wang D.D., Li R., Liu G., Eliassen H.A., Chan A.T. (2022). Dietary lignans, plasma enterolactone levels, and metabolic risk in men: Exploring the role of the gut microbiome. BMC Microbiol..

[84] Xiao H.-H., Zhu Y.-X., Lu L., Zhou L.-P., Poon C.C.-W., Chan C.-O., Wang L.-J., Cao S., Yu W.-X., Wong K.-Y. (2022). The Lignan-Rich Fraction from Sambucus williamsii Hance Exerts Bone Protective Effects via Altering Circulating Serotonin and Gut Microbiota in Rats. Nutrients.

[85] Santangelo R., Silvestrini A., Mancuso C. (2019). Ginsenosides, catechins, quercetin and gut microbiota: Current evidence of challenging interactions. Food Chem. Toxicol..

[86] Bode L.M., Bunzel D., Huch M., Cho G.-S., Ruhland D., Bunzel M., Bub A., Franz C.M.A.P., Kulling S.E. (2013). In vivo and in vitro metabolism of trans-resveratrol by human gut microbiota. Am. J. Clin. Nutr..

